# A simple and effective machine learning model for predicting the stability of intracranial aneurysms using CT angiography

**DOI:** 10.3389/fneur.2024.1398225

**Published:** 2024-06-19

**Authors:** Sha Luo, Li Wen, Yang Jing, Jingxu Xu, Chencui Huang, Zhang Dong, Guangxian Wang

**Affiliations:** ^1^Department of Radiology, Xinqiao Hospital, The Second Affiliated Hospital of Army Medical University, Chongqing, China; ^2^Huiying Medical Technology Co., Ltd., Beijing, China; ^3^Department of Research Collaboration, R&D Center, Beijing Deepwise & League of PHD Technology Co., Ltd., Beijing, China; ^4^Department of Radiology, People’s Hospital of Chongqing Banan District, Chongqing, China

**Keywords:** unstable intracranial aneurysms, machine learning, CT angiography, radiomics, stability

## Abstract

**Background:**

It is vital to accurately and promptly distinguish unstable from stable intracranial aneurysms (IAs) to facilitate treatment optimization and avoid unnecessary treatment. The aim of this study is to develop a simple and effective predictive model for the clinical evaluation of the stability of IAs.

**Methods:**

In total, 1,053 patients with 1,239 IAs were randomly divided the dataset into training (70%) and internal validation (30%) datasets. One hundred and ninety seven patients with 229 IAs from another hospital were evaluated as an external validation dataset. The prediction models were developed using machine learning based on clinical information, manual parameters, and radiomic features. In addition, a simple model for predicting the stability of IAs was developed, and a nomogram was drawn for clinical use.

**Results:**

Fourteen machine learning models exhibited excellent classification performance. Logistic regression Model E (clinical information, manual parameters, and radiomic shape features) had the highest AUC of 0.963 (95% CI 0.943–0.980). Compared to manual parameters, radiomic features did not significantly improve the identification of unstable IAs. In the external validation dataset, the simplified model demonstrated excellent performance (AUC = 0.950) using only five manual parameters.

**Conclusion:**

Machine learning models have excellent potential in the classification of unstable IAs. The manual parameters from CTA images are sufficient for developing a simple and effective model for identifying unstable IAs.

## Introduction

With the increasing availability and quality of noninvasive imaging modalities, a growing number of intracranial aneurysms (IAs) are being detected (1, 2). Computed tomography angiography (CTA) remains the first-line imaging modality due to its features of being noninvasive, fast, cost-effective and wide availability. Most IAs are asymptomatic, but once ruptured, they can lead to subarachnoid hemorrhage (SAH). The mortality and morbidity of aneurysmal SAH are high worldwide, and the 1-year mortality rate can reach 65% (untreated), while approximately half of the survivors are left with permanent neurological deficits (3, 4). Prophylactic treatment of unruptured IAs via endovascular therapy (EVT) or neurosurgical therapy (NST) can decrease the risk of SAH. However, a systemic review revealed that the clinical complication risk and case fatality rate from EVT were 4.96 and 0.30%, respectively, and those from NST were 8.34 and 0.10%, respectively (5). Therefore, the risk of an unruptured IA treatment should be balanced with the risk of rupture.

The processes leading to IA development and rupture are poorly understood. However, the rupture rate of growing unruptured IAs is significantly increased, and patients with IA growth should be strongly considered for treatment (1, 2, 6, 7). In addition, patients with symptomatic unruptured IAs and a history of aneurysmal SAH also have a significantly increased risk of rupture (1, 7–10). Hence, in clinical work, it is vital to accurately and promptly distinguish unstable from stable IAs to facilitate treatment optimization and avoid unnecessary treatment.

**Figure 1:**
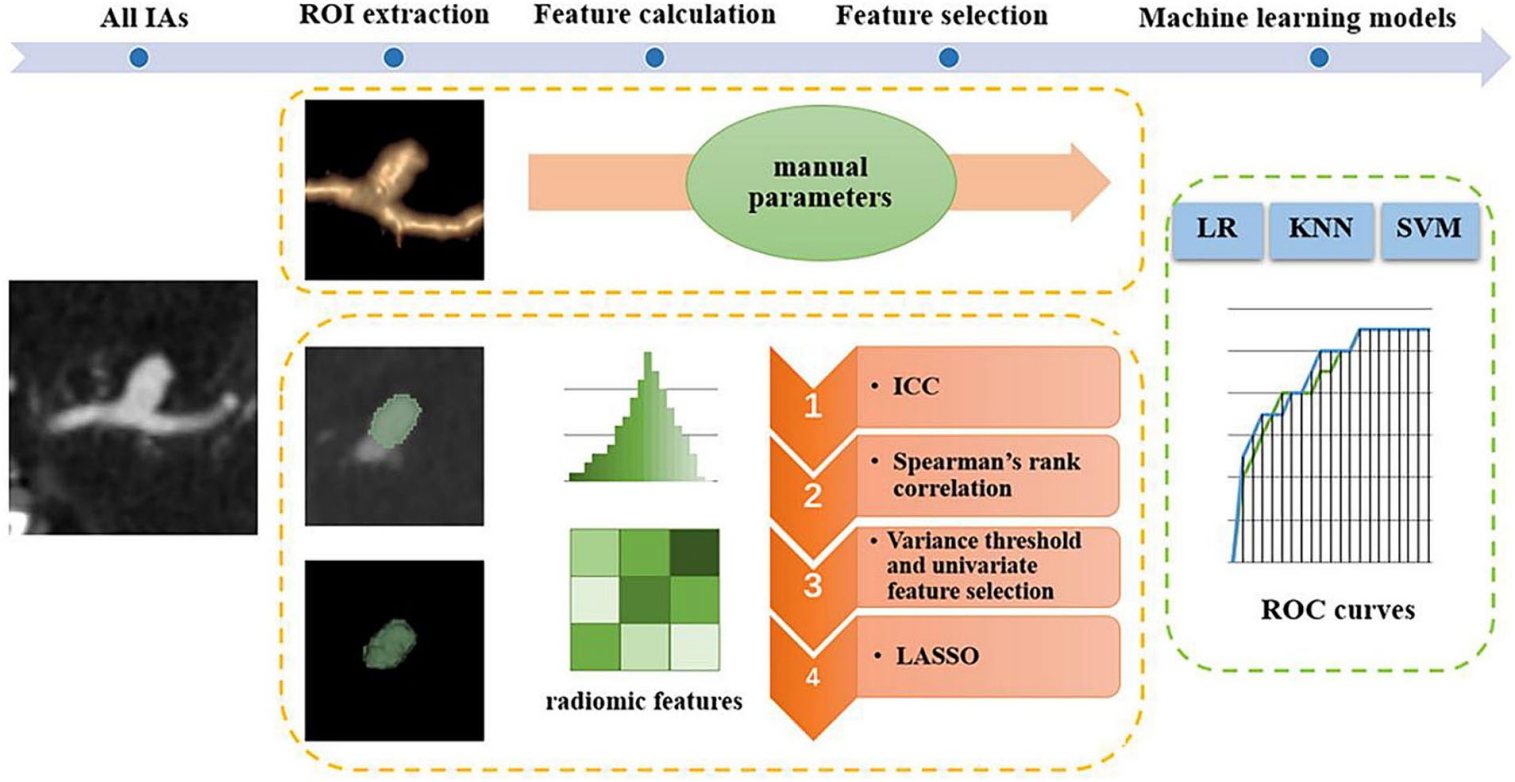
Main steps of the study framework. IAs, intracranial aneurysms; ROI, region of interest; ICC, intraclass correlation coefficient; LASSO, least absolute shrinkage selection operator; ROC, receiver operating characteristic; LR, logistic regression; KNN, k-nearest neighbors; SVM, support vector machine.

In recent years, machine learning and radiomics-based studies have been used to identify risk factors for IA. Furthermore, various rupture and instability classification models for IAs have been proposed (11–18). These studies indicate that machine learning models may help identify potentially ruptured IAs. However, only a few studies have constructed models for the stability of IAs (11, 13). Hence, the purpose of this study was to develop a simple and effective classification model using machine learning based on patient clinical information and CTA images to assist in the clinical evaluation of the stability of IAs. The main steps of this study framework are shown in Figure 1.

## Materials and methods

### Patients

This retrospective study was approved by our institutional ethics committees, and the requirement for informed consent was waived. We retrospectively reviewed the medical records and imaging data in our hospitals from August 2011 to May 2021. The inclusion criteria were as follows: (1) aged older than 18 years, (2) had a diagnosis of saccular IA, and (3) had accessible clinical and radiological data. The exclusion criteria were as follows: (1) a diagnosis of an infectious, traumatic, fusiform, or dissecting IAs; (2) combined with other vascular diseases (e.g., moyamoya disease and arteriovenous malformations); and (3) absence of clinical data or high-quality radiological data and surgery or interventional therapy prior to the CTA examination.

All IAs met the aforementioned inclusion criteria and were divided into 2 groups: stable IAs and unstable IAs. The criteria for unstable IAs included (1) a history of SAH (ruptured but untreated), (2) rupture, growth or gross evolution (e.g., formation of blebs and lobes) during follow-up, and (3) neurologic symptoms (e.g., sudden headache or blepharoptosis) related to the IA. The ruptured IA was confirmed based on operative findings, angiography, or hemorrhage patterns of nonenhanced CT. When patients with multiple IAs and clinical symptoms, but could not determine which IA caused the symptoms, ruling out other causes of symptoms, the largest IA was then identified as the responsible IA for the patients’ neurological symptoms (1, 7–10, 19, 20). For IAs discovered by chance, stable IAs were diagnosed by follow-up of ≥3 months using magnetic resonance angiography (MRA) or CTA (11, 13). Clinical information, such as hypertension or diabetes mellitus, were collected. Finally, a total of 1,053 patients with 1,239 IAs (683 unstable, 556 stable) were retrospectively enrolled to develop classification models, and 197 patients from another hospital with 229 IAs (106 unstable, 123 stable) were used as the external validation set.

### Acquisition of manual parameters

All the CTA images were acquired from a 64-channel multidetector CT scanner with a section thickness of 0.625 mm and a reconstruction interval of 0.625 mm. The images were subsequently transferred to a GE Advantix workstation (Advantage Windows 4.5) to generate 3D volume renderings (VRs) and maximum intensity projections (MIPs). Manual parameters were measured directly from 3-dimensional CTA images according to previous definitions (19, 20). All the images were examined by two experienced neuroradiologists. Continuous data were calculated as the means, while any differences in categorical data were reassessed by a third reader (an observer with 20 years of experience in neuroradiology) for subsequent statistical analysis. And Inter-rater interclass correlation (ICC) was used to evaluate the inter-observer reproducibility.

Twenty-one manually identified and measured parameters (called manual parameters herein) were obtained from the CTA images of each IA: multiplicity; location, which was divided into 6 categories [internal carotid artery (ICA), middle cerebral artery (MCA), anterior cerebral artery (ACA), anterior communicating artery (ACoA), posterior communicating artery (PCoA), and posterior circulation artery (PCA)]; IA size (neck width, depth, width, height, maximum size); IA morphology (irregular shape, which was defined as one that was not smooth or that presented with lobular or daughter sac); and 8 secondary geometric morphology indices: aspect ratio (AR, depth/neck width), AR1 (height/neck width), size ratio (SR, depth/parent artery diameter), SR1 (depth/mean artery diameter), SR2 (maximum size/mean artery diameter), SR3 (maximum size/parent artery diameter), depth-to-width ratio (DW, depth/width) and bottleneck factor (BF, width/neck width); IA origin (sidewall or bifurcation); and parameters related to the parent artery, including the parent artery diameter, mean artery diameter and the flow angle (FA) (13, 19, 20).

### Radiomic feature extraction

Each IA was defined as a region of interest (ROI) and manually segmented by 2 experienced neuroradiologists who were aware of the location of the IA on each slice of the CTA images. Then, 30 aneurysms were randomly selected to be re-segmented by another neuroradiologist. We calculated the inter-rater interclass correlation ICC to evaluate the inter-observer reproducibility. All ROIs were segmented slice by slice on three orthogonal views using the Dr. Wise Multimodal Research Platform.^1^ Subsequently, 2,107 radiomic features were automatically extracted for each ROI using the open-source “PyRadiomics” Python package (21). In this study, radiomics features were divided into two parts: 14 radiomic shape features and a large number of radiomic non-shape features. The radiomic non-shape features were as follows: 18 first-order features, 22 gray-level cooccurrence matrix (GLCM) features, 16 gray-level size zone matrix (GLSZM) features, 16 gray-level run length matrix (GLRLM) features, 14 gray-level dependence matrix (GLDM) features, 5 neighboring gray-tone difference matrix (NGTDM) features, and a total of 2002 higher-order features. These features were calculated from the above features using filters (including exponent, gradient transform, wavelet, Laplace-Gauss, square root, logarithm, and local binary mode transform). All radiomic features were standardized by z score transformation to eliminate the unit limits of each feature. All the calculation equations and the pipeline can be found at https://pyradiomics.readthedocs.io/en/latest/.

### Statistical analysis

#### Clinical information and manual parameters

First, patients with a total of 1,239 IAs (683 unstable, 556 stable) were randomly sampled into a training set and an internal validation set at a 7:3 ratio. Thus, 867 IAs (478 unstable and 389 stable) were randomly selected as the training set for feature selection and classification model construction. The remaining 372 IAs (205 unstable, 167 stable) composed the internal validation set, which was only used to test the model. In addition, an external validation set (106 unstable, 123 stable) was used to ultimately evaluate the effectiveness of the model.

R software (version 4.0.3; Boston, MA, USA) was used in the analyses for all clinical information and manual parameters. Categorical variables are expressed as frequencies, and the Chi-square test was used to evaluate the differences between groups. Continuous variables are expressed as the means. The Shapiro–Wilk test was used to assess the normality of the distribution. The Mann–Whitney *U* test or two-tailed independent Student’s *t* test was used to assess continuous variables as appropriate. Then, only statistically significant variables at the *p* < 0.05 level in univariate analysis were selected as input variables for the machine learning models for all clinical information. The variance threshold (threshold = 0.8), and the least absolute shrinkage selection operator (LASSO) were used to screen for the optimal manual parameters. If the predictive model contained too many parameters, a maximum of 10 parameters with the highest weight were retained. Similar methods were used to screen the radiomic shape features.

The radiomic non-shape features selection steps were as follows: (1) The interrater reliability of the morphology measurements was assessed by the intraclass ICC. Features with an ICC > 0.8 were selected for further analysis. (2) Spearman’s rank correlation was employed to eliminate high-dimensional feature redundancy (for the remaining features after removing 3D-based shape features). If the correlation coefficient was >0.9 for two features, then one of the features was excluded; (3) the variance threshold was set to 0.8 was used; (4) and the LASSO was used and regularized by 10-fold cross-validation to ensure the robustness of the results. Fourteen 3D-based radiomic shape features were filtered out via LASSO regression. Moreover, the 10 features with the highest weights were selected from the other features filtered by LASSO regression (if there were not 10 features in the final LASSO result, all were selected) as the radiomics signature, which was incorporated into the predictive models built using machine learning algorithms.

#### Machine learning model development and evaluation

Four classification models, Model A (manual parameters), Model B (manual parameters + radiomic shape features), Model C (radiomic non-shape features), and Model D (manual parameters + radiomic non-shape features) were developed to identify unstable and stable IAs. Three machine learning algorithms were used to find the optimal classifier for stable and unstable IA classification: logistic regression (LR), k-nearest neighbors (KNN), and support vector machine (SVM) classifiers; these algorithms are widely used in clinical classification and prediction research and have shown good performance (15, 16, 19). The model with the highest classification performance among Models A-D was subsequently reconstructed by adding useful clinical information to obtain Model E. The performance of all the models was assessed by using the area under the receiver operating characteristic (ROC) curve (AUC), accuracy, precision, sensitivity and specificity. The performance of all the models was evaluated in a separate internal validation set and an external validation set.

#### Correlation analysis

Canonical correlation analysis (CCA) is a statistical method used to address the relationship between two random vectors and has been widely used in data analysis, information fusion and other fields (22). CCA was used to explore the associations between the radiomic features of the radiomics signature and manual parameters.

## Results

### Clinical information and manual parameters

Table 1 shows the clinical information and manual parameters of the IAs. The results showed that patients with unstable IAs were younger than were those with stable IAs (*p* = 0.001). Hypertension, heart disease, diabetes mellitus and cerebral vascular sclerosis were more prevalent in the stable group (*p* = 0.012, *p* < 0.001, *p* = 0.001, and *p* < 0.001, respectively).

**Table 1:** Clinical information and manual parameters of the IAs.

Clinical information	Training set		p	Training set	Internal validation set	p
	Stable (*n* = 389)	Unstable (*n* = 478)		(*n* = 867)	(*n* = 372)	
Female (%)	244 (62.7)	306 (64.0)	0.748	550 (63.4)	246 (66.1)	0.4
Age (median [IQR])	61.00 [50.00, 70.00]	57.00 [49.00, 67.00]	0.001	59.00 [49.50, 68.00]	59.00 [49.00, 68.00]	0.929
Hypertension (%)	189 (48.6)	194 (40.6)	0.012	383 (44.2)	172 (46.2)	0.296
Heart disease (%)	47 (12.1)	24 (5.0)	<0.001	71 (8.2)	31 (8.3)	0.999
Diabetes mellitus (%)	42 (10.8)	23 (4.8)	0.001	65 (7.5)	21 (5.6)	0.292
Cerebral vascular sclerosis (%)	86 (22.1)	45 (9.4)	<0.001	131 (15.1)	55 (14.8)	0.952
Alcohol consumption (%)	76 (19.5)	102 (21.3)	0.229	178 (20.5)	67 (18)	0.538
Smoking (%)	92 (23.7)	119 (24.9)	0.736	211 (24.3)	88 (23.5)	0.988
Manual parameters						
Multiple aneurysms (%)	137 (35.2)	101 (21.1)	<0.001	238 (27.5)	109 (29.3)	0.551
Location (%)			<0.001			0.333
ACoA	27 (6.9)	137 (28.7)	<0.001	164 (18.9)	57 (15.3)	0.13
ACA	11 (2.8)	31 (6.5)	0.013	42 (4.8)	15 (4.0)	0.532
MCA	76 (19.5)	75 (15.7)	0.137	151 (17.4)	58 (15.6)	0.432
PCoA	52 (13.4)	158 (33.1)	<0.001	210 (24.2)	106 (28.5)	0.114
ICA	215 (55.3)	56 (11.7)	<0.001	271 (31.3)	119 (32.0)	0.799
PCA	8 (2.1)	21 (4.4)	0.057	29 (3.3)	17 (4.6)	0.296
Neck width (mm)	4.09 (1.15)	5.06 (2.28)	<0.001	4.63 (1.92)	4.52 (1.78)	0.373
Height (mm)	3.25 (1.16)	6.62 (3.67)	<0.001	5.11 (3.29)	4.93 (3.12)	0.382
Depth (mm)	3.44 (1.27)	7.22 (3.87)	<0.001	5.52 (3.54)	5.35 (3.36)	0.418
Width (mm)	3.67 (1.31)	6.54 (4.08)	<0.001	5.25 (3.46)	5.10 (3.34)	0.467
Maximum size (mm)	4.49 (1.46)	8.48 (4.28)	<0.001	6.69 (3.87)	6.55 (3.77)	0.564
Parent artery diameter (mm)	4.02 (0.92)	3.33 (0.86)	<0.001	3.64 (0.95)	3.64 (0.93)	0.945
Mean artery diameter (mm)	3.73 (0.88)	3.02 (0.81)	<0.001	3.33 (0.91)	3.36 (0.93)	0.693
AR	0.85 (0.24)	1.47 (0.56)	<0.001	1.19 (0.54)	1.21 (0.65)	0.588
AR1	0.80 (0.23)	1.35 (0.53)	<0.001	1.10 (0.50)	1.12 (0.62)	0.59
DW	0.96 (0.22)	1.19 (0.37)	<0.001	1.08 (0.33)	1.09 (0.36)	0.938
BF	0.89 (0.18)	1.29 (0.51)	<0.001	1.11 (0.45)	1.12 (0.46)	0.833
SR	0.96 (0.38)	2.46 (1.23)	<0.001	1.79 (1.21)	1.76 (1.43)	0.743
SR1	0.89 (0.35)	2.25 (1.20)	<0.001	1.64 (1.14)	1.62 (1.32)	0.806
SR2	1.26 (0.45)	2.90 (1.35)	<0.001	2.16 (1.33)	2.15 (1.63)	0.94
SR3	1.16 (0.41)	2.64 (1.32)	<0.001	1.97 (1.25)	1.98 (1.54)	0.955
Irregular shape (%)	25 (6.4)	315 (65.9)	<0.001	340 (39.2)	140 (37.6)	0.645
Daughter sac (%)	12 (3.1)	211 (44.1)	<0.001	223 (25.7)	97 (26.1)	0.952
Bifurcation (%)	60 (15.4)	133 (27.8)	<0.001	193 (22.3)	69 (18.5)	0.142
FA (°)	104.33 (28.81)	120.55 (25.49)	<0.001	113.27 (28.19)	111.03 (28.57)	0.201

ICA, internal carotid artery; MCA, middle cerebral artery; ACA, anterior cerebral artery; ACoA, anterior communicating artery; PCoA, posterior communicating artery; PCA, posterior circulation artery; AR, aspect ratio (depth/neck width); SR, size ratio (depth/parent artery diameter); DW, depth-to-width ratio (depth/width); BF, bottleneck factor (width/neck width); IA origin, (sidewall or bifurcation); FA, flow angle.

For manual parameters, the mean Inter-rater ICC value was 0.81. All the manual parameters were significantly associated with IA stability (*p* < 0.001). Multiple IAs were more common in the stable group than in the unstable group (35.2% vs. 21.1%). IA stability was significantly associated with location: unstable IAs were significantly more common than stable IAs in the ACA, ACoA and PCoA (*p* = 0.013, *p* < 0.001, and *p* < 0.001, respectively). In contrast, stable IAs were more prevalent than unstable IAs in the ICA (*p* < 0.001). The sizes (neck width, depth, width, height, maximum size) of the stable IAs were smaller than those of the unstable IAs. Irregular shape, daughter sac and bifurcation type were more common in the unstable group than in the stable group. Moreover, the 8 secondary geometric morphology indices (AR, AR1, DW, BF, SR, SR1, SR2, and SR3) of the unstable IAs were greater than those of the stable IAs. The 10 highest weighted manual parameters are shown in Supplementary Table S1. IA stability was also significantly associated with parameters related to the parent artery. The parent artery diameter and mean artery diameter were greater in the stable than in the unstable IAs. Moreover, there were no significant differences between the training set and the internal validation set. For more information of the IAs in the external validation set, please refer to Supplementary Table S2.

### Optimal radiomics signature

A total of 1,698 radiomic features and 12 radiomic shape features showed high interobserver agreement (ICC > 0.8). Finally, 10 radiomic features and 4 radiomic shape features remained after LASSO regression, as shown in the Supplementary Tables S3, S4. The coefficient–lambda graph and error–lambda graph was shown in Supplementary Figure S1.

### Machine learning models

**Figure 2:**
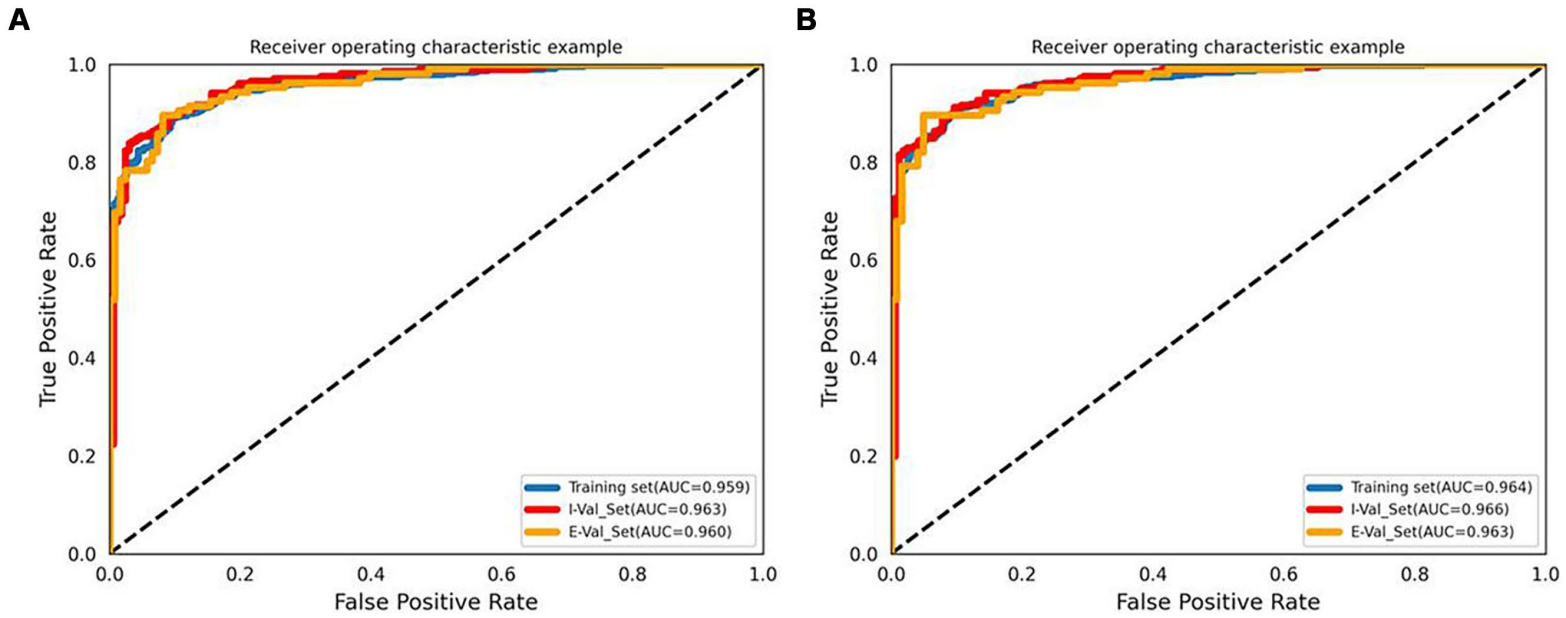
ROC curves and AUCs of the LR Model B **(A)** and Model E **(B)**. I-Val set, internal validation set; E-Val set, external validation set.

The accuracy, precision, sensitivity, and specificity of each model are listed in Supplementary Tables S5–S7. The performance of each model in the external validation set is listed in Table 2. With respect to the external validation set, the results showed that all the machine learning models had outstanding classification ability (all AUCs >0.80). Model B (built by manual parameters and radiomic shape features) showed the best classification performance among models A-D. At the same time, among the four models, the AUC of the LR algorithm was the highest. The AUC values of Model B for the LR, SVM, and KNN algorithms were 0.960 (95% CI 0.939–0.978), 0.952 (95% CI 0.925–0.973), and 0.930 (95% CI 0.900–0.956), respectively. Then, we constructed Model E by adding 5 clinical characteristics, 10 manual parameters, and 4 radiomic shape features, and LR was used to develop this model. LR Model E had an AUC of 0.963 (95% CI 0.943–0.980), an accuracy of 91.7%, a precision of 92.2%, a sensitivity of 89.6%, and a specificity of 93.5%. The ROC curve and AUC of LR Model B and Model E are shown in Figure 2. Nineteen variables in Model E and their importance are shown in Table 3. The *p*-values from the DeLong test of the statistical comparison of the ROC curves in external validation set are given in Supplementary Table S8. The confusion matrix for Model E in the external validation set is shown in Supplementary Figure S2.

**Table 2:** The performance of each model in the external validation set.

Model	Algorithm	AUC	Accuracy	Precision	Sensitivity	Specificity
Model A	LR	0.959 (0.937–0.978)	0.895	0.860	0.925	0.870
	KNN	0.913 (0.879–0.944)	0.852	0.781	0.943	0.772
	SVM	0.947 (0.919–0.971)	0.891	0.852	0.925	0.862
Model B	LR	0.960 (0.939–0.978)	0.891	0.909	0.849	0.927
	KNN	0.930 (0.900–0.956)	0.834	0.793	0.868	0.805
	SVM	0.952 (0.925–0.973)	0.891	0.852	0.925	0.862
Model C	LR	0.899 (0.860–0.933)	0.821	0.788	0.840	0.805
	KNN	0.828 (0.779–0.868)	0.760	0.695	0.858	0.675
	SVM	0.898 (0.855–0.933)	0.834	0.793	0.868	0.805
Model D	LR	0.959 (0.936–0.978)	0.900	0.874	0.915	0.886
	KNN	0.905 (0.871–0.936)	0.834	0.788	0.877	0.797
	SVM	0.942 (0.913–0.967)	0.873	0.829	0.915	0.837
Model E	LR	0.963 (0.943–0.980)	0.917	0.922	0.896	0.935
Simplified model	LR	0.950 (0.924–0.970)	0.869	0.822	0.915	0.929

Model A, manual parameters model; Model B, manual parameters + radiomic shape features model; Model C, radiomics non-shape signature model; Model D, manual parameters + radiomics non-shape signature model; Model E, manual parameters + radiomic shape features + useful clinical information model; LR, logistic regression; KNN, k-nearest neighbors; SVM, support vector machine.

**Table 3:** Nineteen variables and their coefficients in Model E.

Variable	Coefficients
DW	4.221404732
BF	3.013506085
Irregular shape	2.348992875
SR3	0.726773537
Original shape Flatness	0.678361904
Original shape Major Axis Length	0.667566627
Heart disease	0.112164126
Width	0.047364246
Maximum size	0.005060162
Original shape Mesh Volume	0.001738275
Age	−0.012289048
Neck width	−0.013327893
SR1	−0.092761402
Hypertension	−0.181961963
Diabetes mellitus	−0.386633538
Cerebral vascular sclerosis	−1.112285891
original shape Surface Volume Ratio	−1.354612045
SR	−1.966730872
Mean artery diameter	−2.08726665

DW, depth-to-width ratio; BF, bottleneck factor; SR, size ratio.

### The simplified model and nomogram

**Figure 3:**
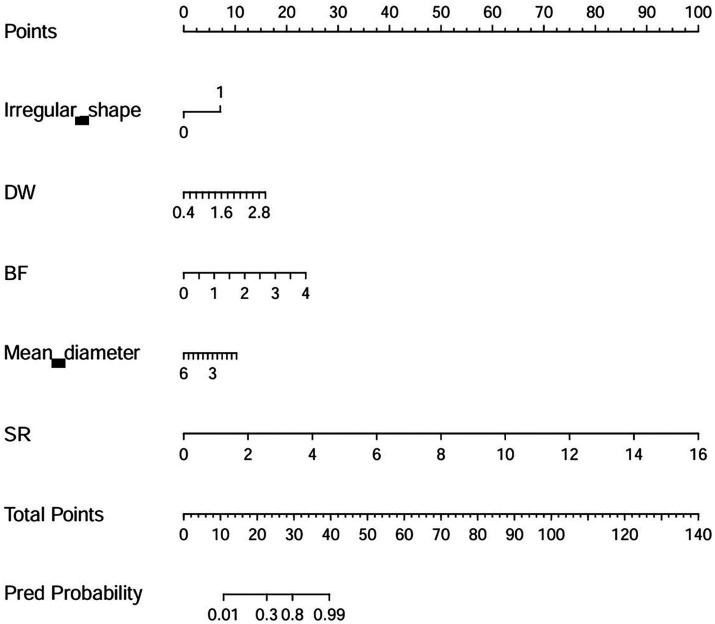
The nomogram of the simplified model. DW, depth-to-width ratio (depth/width); BF, bottleneck factor (width/neck width); SR, size ratio (depth/parent artery diameter).

Model E consists of 19 variables, and too many variables can limit its practical use in clinical settings. Therefore, based on the coefficients (variable importance) of each variable, a simplified model with only 5 manual parameters (absolute coefficients ≥2) was derived, and a nomogram was drawn. The 5 parameters are as follows: irregular shape, DW, BF, mean artery diameter, and SR. The simplified model had an AUC of 0.950 (95% CI 0.924–0.970), an accuracy of 86.9%, a precision of 82.2%, a sensitivity of 91.5%, and a specificity of 82.9%. The DeLong test showed that there is no statistical difference between the Model E and simplified model (*p* = 0.245, in the external validation set). The nomogram of the simplified model is shown in Figure 3.

#### Correlation analysis

The CCA analysis results showed that there was a strong correlation between the radiomic features of the radiomics signature and manual parameters, with a correlation coefficient of 0.822.

## Discussion

In this study, we constructed multiple machine learning models for unstable and stable IAs and evaluated their classification performance; all the machine learning models were found to exhibit good classification ability. The simplified model demonstrated excellent performance (AUC = 0.950) using only five manual parameters.

Machine learning and radiomics are powerful tools that can increase the power of decision support models (23). In this study, our results indicated that all the machine learning models had great potential in classifying unstable IAs. Further analysis of the external validation set revealed that the LR algorithm was superior to the SVM and KNN algorithms among all the models. Similarly, a Chinese multicenter study showed that the KNN and SVM methods cannot outperform conventional LR in prediction models for ruptured IAs (24, 25). Interestingly, the AUC of the LR algorithm was not optimal in the training set or internal validation set; however, the final external validation results were better than those of the KNN and SVM algorithms. This highlights the importance of external validation and the potential advantages of LR.

Currently, most IA studies that apply both machine learning and radiomics have built and assessed classification models for ruptured or unruptured IAs (12, 14, 17, 18). However, simple classification of IAs based on ruptured or unruptured cases is no longer applicable. The rupture rate of growing unruptured IAs significantly increases, and enlargement should replace rupture as an indicator of IA risk (1, 2, 6, 7). Therefore, a portion of unruptured IAs are unstable. For IAs, grouping them based on stability seems to have more clinical significance. This may also explain why the stability classification models of IAs exhibited excellent performance in this study.

To date, few studies have used machine learning to classify IA stability (11, 13). Both of these studies used 3D digital subtraction angiography images. In these studies, the criteria for an unstable IA included rupture within 1 month and growth on sequential imaging follow-up (11, 13). However, growth or overall evolution is not necessarily linear and is sometimes random and discontinuous (2). Some IAs that take longer than 1 month to rupture are also unstable. Thus, given the potentially catastrophic consequences of misdiagnosing unstable IAs as stable with a low risk of rupture, the classification of unstable IAs in this study may be more favorable for patients.

The simplified model also demonstrated excellent classification performance and used only 5 manual parameters, which are the top 5 variables in Model E. Notably, although Model A’s classification ability is slightly lower than that of Model B, Model A only uses manual parameters from CTA images. This means that the parameters from CTA images are sufficient to construct a classification model for identifying unstable IAs. Theoretically, manually measured diameters are semi objective because the results may differ among different raters, and these indices are insufficient for delineating the overall morphological features of the IA. However, manual delineation of ROIs for extracting radiomic features is also currently needed. Furthermore, the rupture risk of an IA and the treatment strategy used are closely related to the IA itself and its parent artery (such as location, flow angles, and bifurcation) (1, 4, 26). For example, for medium and small IAs, the size of the neck is related to the choice of surgical method (27, 28). However, delineating the IA itself as the only ROI would eliminate all parent artery information, and the location and size of the IA neck would not be identified. Our simplified model with only 5 manual parameters (irregular shape, DW, BF, mean artery diameter, and SR) contains important information about the IA and its parent artery. Determine the morphological parameters related to IA rupture by measuring them the risk of rupture of this IA is the ultimate goal of morphological measurement. Our research results also fit this purpose perfectly.

### Limitations

This study has several limitations. First, although ruptured IAs were indeed unstable and a previous study reported that unruptured IAs do not shrink when they rupture (29), post-rupture morphology should not be considered an adequate surrogate for pre-rupture morphology, which may generate a possible bias in our results (30). Second, the IA size was not further graded in this study. The measurement and delineation of ROIs for small IAs may be debatable depending on different observers. Third, incidentally discovered IAs with no apparent symptoms are classified as stable, which may lead to misclassification of some unstable IAs. In the future, our aim is to develop a decision-making tool for use in practical clinical environments and to further validate the large number of enrolled patients using prospective, multicenter designs before use in a real clinical setting.

## Conclusion

Machine learning models have excellent potential in the classification of unstable IAs. This approach will facilitate the rapid and accurate identification of unstable IAs in clinical practice. Compared to manual parameters, radiomic features did not significantly improve the identification of unstable IAs, and the manual parameters from CTA images are sufficient for developing a simple and effective model for identifying unstable IAs. The accuracy of this simple model needs to be further verified through the use of additional data and randomized controlled studies with multicenter data.

## Data availability statement

The original contributions presented in the study are included in the article/Supplementary material, further inquiries can be directed to the corresponding author.

## Ethics statement

The studies involving humans were approved by the Institutional Ethics Committee of Banan hospital and Xinqiao hospital. The studies were conducted in accordance with the local legislation and institutional requirements. The ethics committee/institutional review board waived the requirement of written informed consent for participation from the participants or the participants’ legal guardians/next of kin because this is a retrospective study.

## Author contributions

SL: Data curation, Investigation, Writing – original draft. LW: Data curation, Investigation, Writing – review & editing. YJ: Formal analysis, Writing – review & editing. JX: Investigation, Methodology, Writing – review & editing. CH: Investigation, Methodology, Writing – review & editing. ZD: Conceptualization, Resources, Writing – review & editing. GW: Conceptualization, Funding acquisition, Methodology, Writing – review & editing.

## Footnotes

^1^ https://keyan.deepwise.com

## Supplementary material

SUPPLEMENTARY FIGURE S1 The coefficients-lambda graph **(A)** and error-lambda graph **(B)**.

SUPPLEMENTARY FIGURE S2 The confusion matrix for Model E.
