# Supplementary material for: A simple and effective machine learning model for predicting the stability of intracranial aneurysms using CT angiography
Source: Front Neurol. 2024 Jun 19;15:1398225. doi: 10.3389/fneur.2024.1398225 (PMC11219573; doi:10.3389/fneur.2024.1398225)
Supplement: Supplementary file 1 [file Table_1.DOCX]

| Table S1. The highest weighted manual parameters. | |
| --- | --- |
| **Manual parameters** | **coefficients value** |
| Irregular shape | 0.11879349 |
| SR | -0.170136289 |
| SR1 | -0.094477881 |
| SR3 | 0.134934266 |
| Maximum size | -0.077015598 |
| BF | 0.212816783 |
| Width | 0.059307816 |
| DW | 0.222617651 |
| Mean artery diameter | -0.091757139 |
| Neck width | 0.180934415 |
| SR, size ratio; BF, bottleneck factor; DW, depth-to-width ratio. | |
