# Supplementary material for: A simple and effective machine learning model for predicting the stability of intracranial aneurysms using CT angiography
Source: Front Neurol. 2024 Jun 19;15:1398225. doi: 10.3389/fneur.2024.1398225 (PMC11219573; doi:10.3389/fneur.2024.1398225)
Supplement: Supplementary file 2 [file Table_2.DOCX]

| **Table S2** Clinical information and manual parameters of the IAs in external validation set. | | | |
| --- | --- | --- | --- |
| **Clinical information** | **External validation set** | | ***p*** |
|  | **Stable (n=123)** | **Unstable (n=106)** |  |
| Female (%) | 81 (65.9) | 72 (67.9) | 0.848 |
| Age (median [IQR]) | 67.03 (11.53) | 64.41 (12.43) | 0.099 |
| Hypertension (%) | 41 (33.3) | 37 (34.9) | 0.912 |
| Heart disease (%) | 30 (24.4) | 22 (20.8) | 0.619 |
| Diabetes mellitus (%) | 63 (51.2) | 63 (59.4) | 0.266 |
| Cerebral vascular sclerosis (%) | 26 (21.1) | 11 (10.4) | 0.043 |
| Alcohol consumption (%) | 32 (26.0) | 17 (16.0) | 0.094 |
| Smoking (%) | 32 (26.0) | 22 (20.8) | 0.436 |
| SAH history (%) | 0 (0.0) | 1 (0.9) | 0.941 |
| **Manual parameters** |  |  |  |
|  |  |  |  |
| Multiple aneurysms (%) | 27 (22.0) | 27 (25.5) | 0.639 |
| Location (%) |  |  | <0.001 |
| ACoA | 10 (8.1) | 43 (40.6) |  |
| ACA | 3 (2.4) | 2 (1.9) |  |
| MCA | 20 (16.3) | 15 (14.2) |  |
| PCoA | 28 (22.8) | 36 (34.0) |  |
| ICA | 57 (46.3) | 5 (4.7) |  |
| PCA | 5 (4.1) | 5 (4.7) |  |
| Neck width (mm) | 3.67 (1.01) | 5.05 (1.71) | <0.001 |
| Height (mm) | 2.46 (0.96) | 5.48 (2.70) | <0.001 |
| Depth (mm) | 2.60 (1.04) | 5.87 (2.79) | <0.001 |
| Width (mm) | 3.16 (1.03) | 6.34 (3.54) | <0.001 |
| Maximum size (mm) | 3.90 (1.22) | 7.71 (3.34) | <0.001 |
| Parent artery diameter (mm) | 3.86 (0.94) | 3.41 (0.98) | 0.001 |
| Mean artery diameter (mm) | 3.68 (0.93) | 3.09 (0.86) | <0.001 |
| AR | 0.73 (0.28) | 1.21 (0.50) | <0.001 |
| AR1 | 0.69 (0.27) | 1.13 (0.50) | <0.001 |
| DW | 0.83 (0.24) | 1.00 (0.34) | <0.001 |
| BF | 0.86 (0.17) | 1.26 (0.51) | <0.001 |
| SR | 0.74 (0.33) | 2.05 (1.21) | <0.001 |
| SR1 | 0.71 (0.34) | 1.87 (1.13) | <0.001 |
| SR2 | 1.12 (0.43) | 2.66 (1.32) | <0.001 |
| SR3 | 1.07 (0.46) | 2.43 (1.24) | <0.001 |
| Irregular shape (%) | 5 (4.1) | 79 (74.5) | <0.001 |
| Daughter sac (%) | 3 (2.4) | 68 (64.2) | <0.001 |
| Bifurcation (%) | 21 (17.1) | 57 (53.8) | <0.001 |
| FA (°) | 109.16 (28.81) | 133.24 (23.04) | <0.001 |
| IAs, intracranial aneurysms; ICA, internal carotid artery; MCA, middle cerebral artery; ACA, anterior cerebral artery; ACoA, anterior communicating artery; PCoA, posterior communicating artery; PCA, posterior circulation artery; AR, aspect ratio; SR, size ratio; DW, depth-to-width ratio; BF, bottleneck factor; FA, flow angle. | | | |
