# Supplementary material for: A simple and effective machine learning model for predicting the stability of intracranial aneurysms using CT angiography
Source: Front Neurol. 2024 Jun 19;15:1398225. doi: 10.3389/fneur.2024.1398225 (PMC11219573; doi:10.3389/fneur.2024.1398225)
Supplement: Supplementary file 3 [file Table_3.DOCX]

| **Table S3.** Radiomic features remained as the radiomics signature after LASSO regression. | |
| --- | --- |
| **Radiomics signature** | **Coefficients value** |
| log-sigma-2-0-mm-3D_glcm_Idn | 0.106920302 |
| wavelet-HLH_glcm_Imc2 | -0.05960772 |
| log-sigma-2-0-mm-3D_glcm_Imc2 | -0.08230734 |
| wavelet-HLL glszm Zone Percentage | 0.054595139 |
| wavelet-HLL ngtdm Strength | -0.080233512 |
| Exponential glrlm Short Run Low Gray Level Emphasis | 0.058504651 |
| wavelet-LLH ngtdm Strength | -0.080496433 |
| wavelet-LLH firstorder Entropy | 0.073650421 |
| lbp-3D-k_glcm_Joint Energy | 0.079769407 |
| lbp-3D-k_first order_Mean | 0.055315746 |
