# Supplementary material for: A simple and effective machine learning model for predicting the stability of intracranial aneurysms using CT angiography
Source: Front Neurol. 2024 Jun 19;15:1398225. doi: 10.3389/fneur.2024.1398225 (PMC11219573; doi:10.3389/fneur.2024.1398225)
Supplement: Supplementary file 4 [file Table_4.DOCX]

| **Table S4.** Radiomic shape features remained after LASSO regression. | |
| --- | --- |
| **Radiomic shape features** | **Coefficients value** |
| Original shape Flatness | 0.678361904 |
| Original shape Major Axis Length | 0.667566627 |
| Original shape Surface Volume Ratio | -1.354612045 |
| Original shape Mesh Volume | 0.001738275 |
