# Supplementary material for: A simple and effective machine learning model for predicting the stability of intracranial aneurysms using CT angiography
Source: Front Neurol. 2024 Jun 19;15:1398225. doi: 10.3389/fneur.2024.1398225 (PMC11219573; doi:10.3389/fneur.2024.1398225)
Supplement: Supplementary file 5 [file Table_5.DOCX]

| **Table S5.** Performance of each model using the LR algorithm. | | | | | | |
| --- | --- | --- | --- | --- | --- | --- |
| Model | Data Group | AUC | Accuracy | Precision | Sensitivity | Specificity |
| Model A | Training set | 0.949(0.937-0.960) | 0.884 | 0.914 | 0.87 | 0.9 |
|  | internal validation set | 0.955(0.937-0.971) | 0.882 | 0.913 | 0.868 | 0.898 |
|  | external validation set | 0.959(0.937-0.978) | 0.895 | 0.86 | 0.925 | 0.87 |
| Model B | Training set | 0.959(0.949-0.968) | 0.887 | 0.926 | 0.864 | 0.915 |
|  | internal validation set | 0.963(0.947-0.977) | 0.895 | 0.941 | 0.863 | 0.934 |
|  | external validation set | 0.960(0.939-0.978) | 0.891 | 0.909 | 0.849 | 0.927 |
| Model C | Training set | 0.911(0.894-0.926) | 0.826 | 0.856 | 0.822 | 0.83 |
|  | internal validation set | 0.891(0.862-0.918) | 0.815 | 0.824 | 0.844 | 0.778 |
|  | external validation set | 0.899(0.860-0.933) | 0.821 | 0.788 | 0.84 | 0.805 |
| Model D | Training set | 0.957(0.947-0.966) | 0.887 | 0.934 | 0.856 | 0.925 |
|  | internal validation set | 0.957(0.939-0.973) | 0.884 | 0.918 | 0.868 | 0.904 |
|  | external validation set | 0.959(0.936-0.978) | 0.9 | 0.874 | 0.915 | 0.886 |
| Model E | Training set | 0.964(0.954-0.972) | 0.897 | 0.929 | 0.881 | 0.918 |
|  | internal validation set | 0.966(0.951-0.979) | 0.89 | 0.932 | 0.863 | 0.922 |
|  | external validation set | 0.963(0.943-0.980) | 0.917 | 0.922 | 0.896 | 0.935 |
| Model A, manual parameters model; Model B, manual parameters + radiomic shape features model; Model C, radiomics non-shape model; Model D, manual parameters + radiomics non-shape model; Model E, manual parameters + the radiomic shape features + useful clinical information model; LR, logistic regression; AUC, area under the curve. | | | | | | |
