# Supplementary material for: A simple and effective machine learning model for predicting the stability of intracranial aneurysms using CT angiography
Source: Front Neurol. 2024 Jun 19;15:1398225. doi: 10.3389/fneur.2024.1398225 (PMC11219573; doi:10.3389/fneur.2024.1398225)
Supplement: Supplementary file 6 [file Table_6.DOCX]

| **Table S6.** Performance of each model using the KNN algorithm. | | | | | | |
| --- | --- | --- | --- | --- | --- | --- |
| Model | Data Group | AUC | Accuracy | Precision | Sensitivity | Specificity |
| Model A | Training set | 0.974(0.968-0.979) | 0.902 | 0.923 | 0.897 | 0.907 |
|  | internal validation set | 0.906(0.879-0.930) | 0.841 | 0.869 | 0.839 | 0.844 |
|  | external validation set | 0.913(0.879-0.944) | 0.852 | 0.781 | 0.943 | 0.772 |
| Model B | Training set | 0.976(0.970-0.981) | 0.909 | 0.935 | 0.897 | 0.923 |
|  | internal validation set | 0.912(0.888-0.937) | 0.849 | 0.882 | 0.839 | 0.862 |
|  | external validation set | 0.930(0.900-0.956) | 0.834 | 0.793 | 0.868 | 0.805 |
| Model C | Training set | 0.957(0.948-0.965) | 0.886 | 0.886 | 0.91 | 0.856 |
|  | internal validation set | 0.822(0.788-0.856) | 0.774 | 0.776 | 0.829 | 0.707 |
|  | external validation set | 0.828(0.779-0.868) | 0.760 | 0.695 | 0.858 | 0.675 |
| Model D | Training set | 0.980(0.975-0.985) | 0.922 | 0.934 | 0.923 | 0.92 |
|  | internal validation set | 0.912(0.887-0.934) | 0.858 | 0.858 | 0.888 | 0.82 |
|  | external validation set | 0.905(0.871-0.936) | 0.834 | 0.788 | 0.877 | 0.797 |
| Model A, manual parameters model; Model B, manual parameters + radiomic shape features model; Model C, radiomics non-shape model; Model D, manual parameters + radiomics non-shape model; KNN, k-nearest neighbors; AUC, area under the curve. | | | | | | |
