# Supplementary material for: A simple and effective machine learning model for predicting the stability of intracranial aneurysms using CT angiography
Source: Front Neurol. 2024 Jun 19;15:1398225. doi: 10.3389/fneur.2024.1398225 (PMC11219573; doi:10.3389/fneur.2024.1398225)
Supplement: Supplementary file 7 [file Table_7.DOCX]

| **Table S7.** Performance of each model using the SVM algorithm. | | | | | | |
| --- | --- | --- | --- | --- | --- | --- |
| Model | Data Group | AUC | Accuracy | Precision | Sensitivity | Specificity |
| Model A | Training set | 0.949(0.936-0.959) | 0.887 | 0.911 | 0.881 | 0.895 |
|  | internal validation set | 0.955(0.938-0.970) | 0.884 | 0.926 | 0.859 | 0.916 |
|  | external validation set | 0.947(0.919-0.971) | 0.891 | 0.852 | 0.925 | 0.862 |
| Model B | Training set | 0.960(0.948-0.970) | 0.904 | 0.925 | 0.90 | 0.91 |
|  | internal validation set | 0.958(0.941-0.973) | 0.892 | 0.927 | 0.873 | 0.916 |
|  | external validation set | 0.952(0.925-0.973) | 0.891 | 0.852 | 0.925 | 0.862 |
| Model C | Training set | 0.944(0.931-0.956) | 0.869 | 0.886 | 0.874 | 0.861 |
|  | internal validation set | 0.895(0.866-0.920) | 0.828 | 0.837 | 0.854 | 0.796 |
|  | external validation set | 0.898(0.855-0.933) | 0.834 | 0.793 | 0.868 | 0.805 |
| Model D | Training set | 0.978(0.971-0.985) | 0.926 | 0.954 | 0.91 | 0.946 |
|  | internal validation set | 0.950(0.931-0.966) | 0.879 | 0.904 | 0.873 | 0.886 |
|  | external validation set | 0.942(0.913-0.967) | 0.873 | 0.829 | 0.915 | 0.837 |
| Model A, manual parameters model; Model B, manual parameters + radiomic shape features model; Model C, radiomics non-shape model; Model D, manual parameters + radiomics non-shape model; SVM, support vector machine; AUC, area under the curve. | | | | | | |
