# Supplementary material for: A simple and effective machine learning model for predicting the stability of intracranial aneurysms using CT angiography
Source: Front Neurol. 2024 Jun 19;15:1398225. doi: 10.3389/fneur.2024.1398225 (PMC11219573; doi:10.3389/fneur.2024.1398225)
Supplement: Supplementary file 8 [file Table_8.DOCX]

| Table S8. Delong test results for all LR models, in external validation set. | |
| --- | --- |
| Molde Comparation | *P* Value |
| Model A vs Model B | 0.896 |
| Model A vs Model C | 0.004 |
| Model A vs Model D | 0.924 |
| Model C vs Model B | <0.001 |
| Model D vs Model B | 0.777 |
| Model C vs Model D | <0.001 |
| Model E vs Model B | 0.445 |
| Model E vs simplified model | 0.254 |
| Model B vs simplified model | 0.313 |
| LR, logistic regression. | |
