# Supplementary figures and images for: A simple and effective machine learning model for predicting the stability of intracranial aneurysms using CT angiography
Source: Front Neurol. 2024 Jun 19;15:1398225. doi: 10.3389/fneur.2024.1398225 (PMC11219573; doi:10.3389/fneur.2024.1398225)

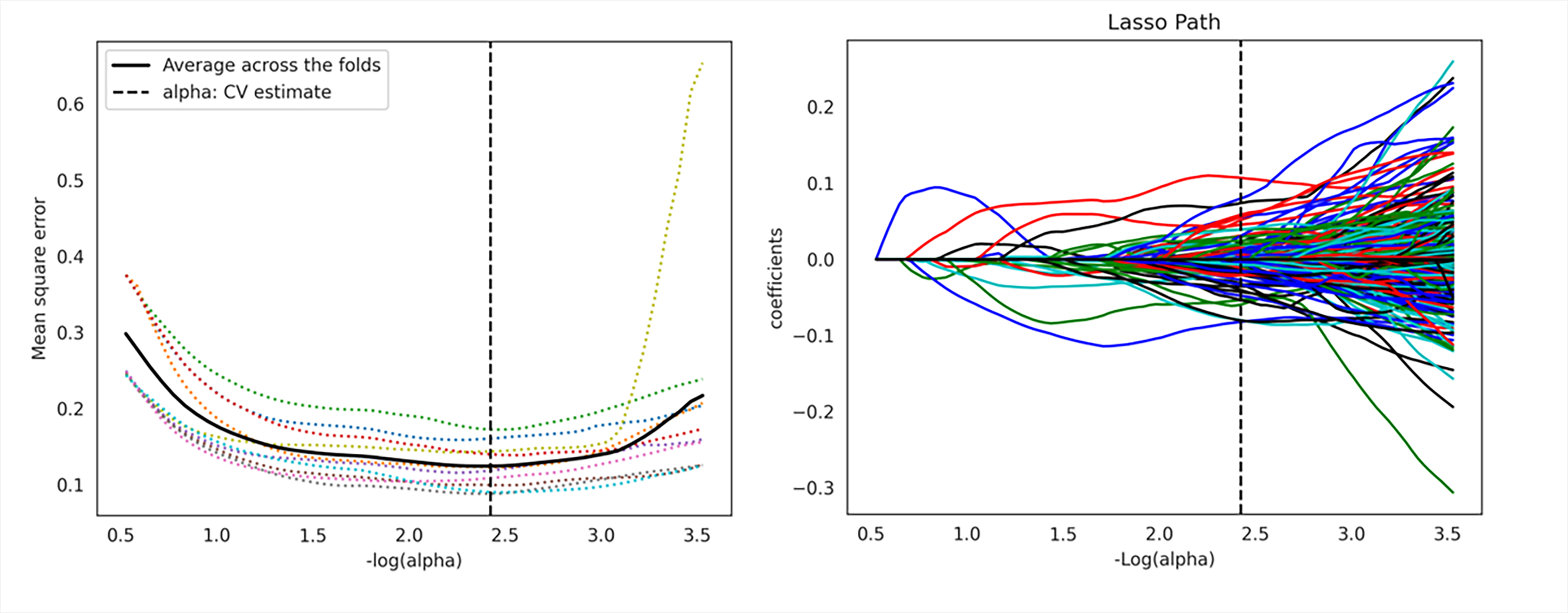

Supplement: SUPPLEMENTARY FIGURE S1 — The coefficients-lambda graph (A) and error-lambda graph (B). [file Image_1.TIF]

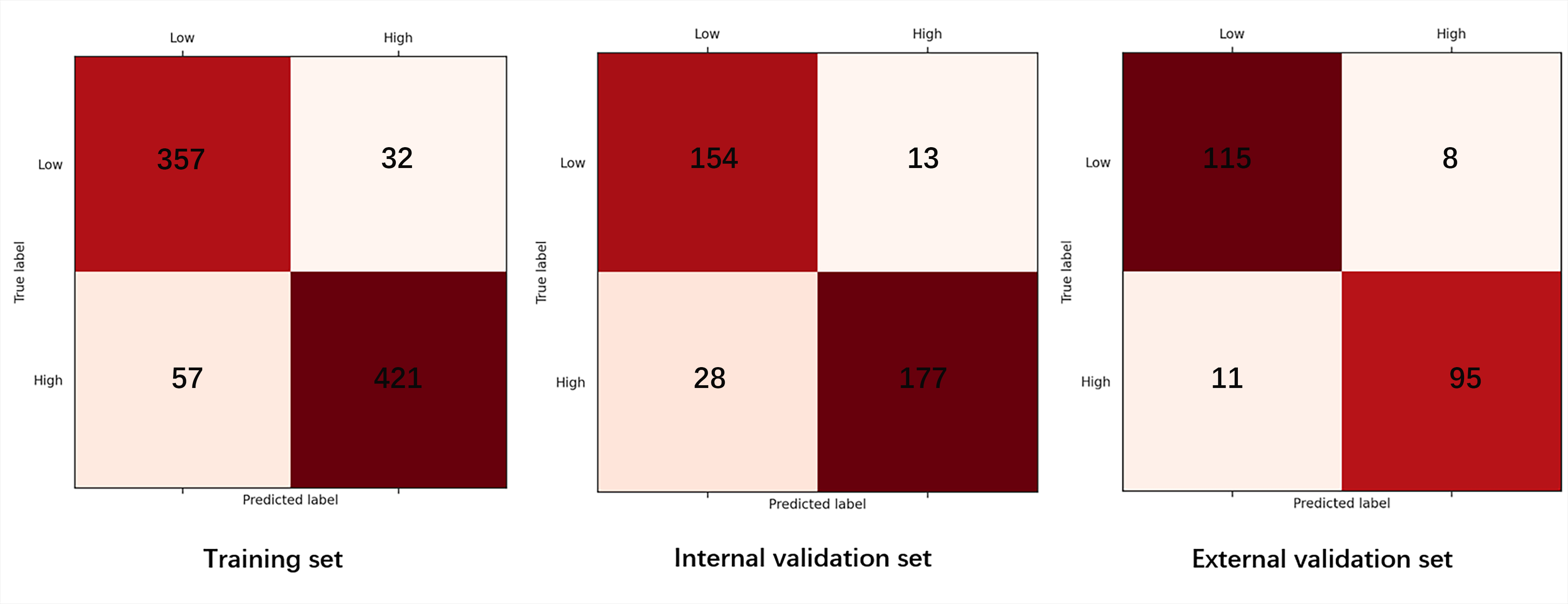

Supplement: SUPPLEMENTARY FIGURE S2 — The confusion matrix for Model E. [file Image_2.TIF]
